# Association between Genetic Variants and Cisplatin-Induced Nephrotoxicity: A Genome-Wide Approach and Validation Study

**DOI:** 10.3390/jpm11111233

**Published:** 2021-11-20

**Authors:** Zulfan Zazuli, Corine de Jong, Wei Xu, Susanne J. H. Vijverberg, Rosalinde Masereeuw, Devalben Patel, Maryam Mirshams, Khaleeq Khan, Dangxiao Cheng, Bayardo Ordonez-Perez, Shaohui Huang, Anna Spreafico, Aaron R. Hansen, David P. Goldstein, John R. de Almeida, Scott V. Bratman, Andrew Hope, Jennifer J. Knox, Rebecca K. S. Wong, Gail E. Darling, Abhijat Kitchlu, Simone W. A. van Haarlem, Femke van der Meer, Anne S. R. van Lindert, Alexandra ten Heuvel, Jan Brouwer, Colin J. D. Ross, Bruce C. Carleton, Toine C. G. Egberts, Gerarda J. M. Herder, Vera H. M. Deneer, Anke H. Maitland-van der Zee, Geoffrey Liu

**Affiliations:** 1 Department of Respiratory Medicine, Academic Medical Centers, University of Amsterdam, 1105 AZ Amsterdam, The Netherlands or zulfan@fa.itb.ac.id (Z.Z.); s.j.vijverberg@amsterdamumc.nl (S.J.H.V.); 2 Department of Pharmacology-Clinical Pharmacy, School of Pharmacy, Bandung Institute of Technology, Bandung 40132, Indonesia; 3 Department of Clinical Pharmacy, St. Antonius Hospital, 3430 EM Nieuwegein, The Netherlands; d.c.dejong-22@umcutrecht.nl; 4 Department of Clinical Pharmacy, Division Laboratories, Pharmacy, and Biomedical Genetics, University Medical Center Utrecht, 3508 GA Utrecht, The Netherlands; a.c.g.egberts@umcutrecht.nl (T.C.G.E.); v.h.m.deneer@umcutrecht.nl (V.H.M.D.); 5 Department of Biostatistics, Dalla Lana School of Public Health, Princess Margaret Cancer Centre, University of Toronto, Toronto, ON M5G 2M9, Canada; wei.xu@uhnresearch.ca; 6 Division of Pharmacology, Utrecht Institute of Pharmaceutical Sciences, Utrecht University, 3584 CG Utrecht, The Netherlands; r.masereeuw@uu.nl; 7 Division of Medical Oncology and Hematology, Department of Medicine, Princess Margaret Cancer Centre, University of Toronto, Toronto, ON M5G 2M9, Canada; devalben.patel@uhn.ca (D.P.); maryam.mirshams@uhn.ca (M.M.); khaleeq.khan@uhnresearch.ca (K.K.); dangxiao.cheng@uhnresearch.ca (D.C.); anna.spreafico@uhn.ca (A.S.); aaron.hansen@uhn.ca (A.R.H.); jennifer.knox@uhn.ca (J.J.K.); 8 Department of Laboratory Medicine and Pathology, University Health Network, University of Toronto, Toronto, ON M5G 2C4, Canada; bayardo.perez-ordonez@uhn.ca; 9 Department of Radiation Oncology, Princess Margaret Cancer Centre, University of Toronto, Toronto, ON M5G 2M9, Canada; shaohui.huang@rmp.uhn.ca (S.H.); scott.bratman@rmp.uhn.ca (S.V.B.); andrew.hope@rmp.uhn.ca (A.H.); rebecca.wong@rmp.uhn.ca (R.K.S.W.); 10 Department of Otolaryngology–Head and Neck Surgery, Princess Margaret Cancer Centre, University of Toronto, Toronto, ON M5G 2M9, Canada; david.goldstein@uhn.ca (D.P.G.); john.dealmeida@uhn.ca (J.R.d.A.); 11 Department of Thoracic Surgery, University Health Network, University of Toronto, Toronto, ON M5G 2C4, Canada; gail.darling@uhn.ca; 12 Department of Medicine, Nephrology, University Health Network, University of Toronto, Toronto, ON M5G 2M9, Canada; abhijat.kitchlu@uhn.ca; 13 Department of Pulmonology, St Antonius Hospital, 3430 EM Nieuwegein, The Netherlands; s.van.haarlem@antoniusziekenhuis.nl; 14 Department of Pulmonology, Diakonessenhuis, 3582 KE Utrecht, The Netherlands; fvdmeer@diakhuis.nl; 15 Department of Pulmonology, University Medical Center Utrecht, 3508 GA Utrecht, The Netherlands; A.S.R.vanLindert-2@umcutrecht.nl; 16 Department of Pulmonology, Groene Hart Hospital, 2803 HH Gouda, The Netherlands; alexandra.ten.heuvel@ghz.nl; 17 Department of Pulmonology, Rivierenland Hospital, 4002 WP Tiel, The Netherlands; jan.brouwer@zrt.nl; 18 British Columbia Children’s Hospital Research Institute, University of British Columbia, Vancouver, BC V5Z 4H4, Canada; colin.ross@ubc.ca (C.J.D.R.); bcarleton@popi.ubc.ca (B.C.C.); 19 Faculty of Pharmaceutical Sciences, University of British Columbia, Vancouver, BC V6T 1Z3, Canada; 20 Division of Translational Therapeutics, Department of Pediatrics, University of British Columbia, Vancouver, BC V1Y 1T3, Canada; 21 Pharmaceutical Outcomes Program, British Columbia Children’s Hospital, Vancouver, BC V5Z 4H4, Canada; 22 Division of Pharmacoepidemiology and Clinical Pharmacology, Utrecht Institute for Pharmaceutical Sciences, Utrecht University, 3584 CG Utrecht, The Netherlands; 23 Meander Medical Center, Department of Pulmonology, 3813 TZ Amersfoort, The Netherlands; GJM.Herder@meandermc.nl; 24 Departments of Medical Biophysics, Pharmacology and Toxicology, and Epidemiology, Dalla Lana School of Public Health and University of Toronto, Toronto, ON M5T 3M7, Canada

**Keywords:** pharmacogenomics, cisplatin, nephrotoxicity, kidney injury, genetic polymorphisms, genome-wide association study, platinum

## Abstract

This study aims to evaluate genetic risk factors for cisplatin-induced nephrotoxicity by investigating not previously studied genetic risk variants and further examining previously reported genetic associations. A genome-wide study (GWAS) was conducted in genetically estimated Europeans in a discovery cohort of cisplatin-treated adults from Toronto, Canada, followed by a candidate gene approach in a validation cohort from the Netherlands. In addition, previously reported genetic associations were further examined in both the discovery and validation cohorts. The outcome, nephrotoxicity, was assessed in two ways: (i) decreased estimated glomerular filtration rate (eGFR), calculated using the Chronic Kidney Disease Epidemiology Collaboration formula (CKD-EPI) and (ii) increased serum creatinine according to the Common Terminology Criteria for Adverse Events v4.03 for acute kidney injury (AKI-CTCAE). Four different Illumina arrays were used for genotyping. Standard quality control was applied for pre- and post-genotype imputation data. In the discovery cohort (*n* = 608), five single-nucleotide polymorphisms (SNPs) reached genome-wide significance. The A allele in rs4388268 (minor allele frequency = 0.23), an intronic variant of the *BACH2* gene, was consistently associated with increased risk of cisplatin-induced nephrotoxicity in both definitions, meeting genome-wide significance (β = −8.4, 95% CI −11.4–−5.4, *p* = 3.9 × 10^−8^) for decreased eGFR and reaching suggestive association (OR = 3.9, 95% CI 2.3–6.7, *p* = 7.4 × 10^−7^) by AKI-CTCAE. In the validation cohort of 149 patients, this variant was identified with the same direction of effect (eGFR: β = −1.5, 95% CI −5.3–2.4, AKI-CTCAE: OR = 1.7, 95% CI 0.8–3.5). Findings of our previously published candidate gene study could not be confirmed after correction for multiple testing. Genetic predisposition of *BACH2* (rs4388268) might be important in the development of cisplatin-induced nephrotoxicity, indicating opportunities for mechanistic understanding, tailored therapy and preventive strategies.

## 1. Introduction

Since its approval by the FDA in 1978 [1], cisplatin has remained a backbone antineoplastic agent used to treat various cancers, such as head and neck, ovarian, testicular, cervical, bladder, gastroesophageal, breast and lung cancer [2,3]. Cisplatin binds to the N7 reactive center on purine residues after entering the cell and exerts its cytotoxic effects via DNA damage in cancer cells, blocking cell division and resulting in apoptotic cell death [2]. Cisplatin also causes endoplasmic reticulum and mitochondria dysfunction [4]. However, its effectiveness also coincides with numerous acute and long-term adverse effects [4,5] such as bone marrow suppression, nausea and vomiting, nephrotoxicity, ototoxicity, and neurotoxicity, which may hamper the antineoplastic potential for the individual patient [2,6].

Approximately one-third of patients develop any kind of nephrotoxicity after a single dose of 50–100 mg/m^2^ cisplatin [7], while up to 90% of patients experience hypomagnesemia, which may exacerbate cisplatin nephrotoxicity, if no corrective measures are initiated [8]. Clinically, nephrotoxicity can lead to various renal manifestations such as acute kidney injury, hypomagnesemia, distal renal tubular acidosis, hypocalcemia, renal salt wasting, renal concentrating defect, hyperuricemia, transient proteinuria, erythropoietin deficiency, thrombotic microangiopathy, and chronic kidney disease (CKD) [9]. Ultimately, CKD may result in significantly elevated cardiovascular mortality risk and further preclude patients from subsequent cisplatin or other cancer therapies [10]. Four potential mechanisms of cisplatin-induced nephrotoxicity have been suggested [11]: (1) proximal tubular injury, (2) oxidative stress, (3) inflammation, and (4) vascular injury in the kidney. Strategies to prevent cisplatin-induced nephrotoxicity are commonly applied in clinical settings, including intravenous fluid repletion with or without magnesium supplementation and mannitol forced diuresis in select patients [12]. However, the risk of kidney damage remains to a significant extent.

Non-genetic risk factors for cisplatin-induced nephrotoxicity have been identified, including older age, low functional status, malnourishment, hypovolemia, presence of chronic comorbid illnesses (e.g., vascular disease, diabetes mellitus, and liver dysfunction), pre-existing (chronic) kidney disease, concurrent nephrotoxic drug exposure (e.g., iodinated contrast, chronic use of non-steroid anti-inflammation drugs (NSAIDs), and gemcitabine), electrolyte disturbances (low serum magnesium levels), alcohol ingestion, and high cisplatin doses per administration (≥50 mg/m^2^), greater frequency of administration, greater cumulative dose, and insufficient intravenous fluid during cisplatin administration [4]. However, studies that have investigated genetic contributions to the development of cisplatin-induced nephrotoxicity have shown inconsistent findings, potentially due to significant patient and treatment heterogeneity along with variability in candidate gene study designs [13]. Nevertheless, a variation in *SLC22A2* rs316019, a gene involved in platinum uptake by the kidney, was associated with different nephrotoxicity definitions in four independent candidate gene studies [13]. Furthermore, variants of *ERCC1* (rs11615 and rs3212986) and *ERCC2* (rs13181 and rs1799793), two genes involved in DNA repair, were found to be associated with increased risks of nephrotoxicity in two independent candidate gene studies [13,14,15,16]. At this stage, a genome-wide approach is preferred to identify unreported genetic associations as well as to confirm previous reported findings. Compared to the candidate gene approach, genome-wide association studies (GWASs) offer an unbiased method to identify genetic variants through scanning of the genome. This includes the identification of novel causal genetic variants providing an opportunity to improve mechanistic understanding of cisplatin-induced nephrotoxicity [17,18,19]. To our knowledge, to date, only candidate gene studies and not GWASs have been performed to evaluate genetic risk factors for cisplatin-induced nephrotoxicity.

In addition, understanding the potential contribution of genetic variants in the occurrence of cisplatin-induced nephrotoxicity could help physicians identify individuals at risk of nephrotoxicity and may assist in guiding optimal drug and dose selection and preventive strategies. Utilizing patients’ genetic information could thus enable safer, more effective, and more cost-effective treatment [20].

This study evaluated the relationship between genetic risk factors and cisplatin-induced nephrotoxicity by investigating genetic risk variants not previously studied through the use of GWAS. An independent validation cohort using a candidate gene approach was used to confirm genetic variations (single-nucleotide polymorphisms (SNPs)) associated with the risk of cisplatin-induced nephrotoxicity from the GWAS. In addition, previously reported genetic associations were further examined in both the discovery and validation cohorts.

## 2. Materials and Methods

### 2.1. Study Design and Patients

#### 2.1.1. Discovery Cohort

A retrospective analysis was performed in a discovery cohort, which consisted of two groups of patients newly diagnosed with head and neck cancer and one group of patients diagnosed with esophageal cancer, all of whom were treated at Princess Margaret Cancer Centre in Toronto, Canada between July 2002 and December 2017. The inclusion criteria for patients in the discovery cohort were as follows: (1) ≥18 years of age, (2) had received high-dose (≥75 mg/m^2^) cisplatin administered in three-week intervals for at least one cycle, either as a single agent or in combination with either other antineoplastic agents and/or radiation for curative intent, (3) estimated glomerular filtration rate (eGFR) ≥ 60 mL/min/1.73 m^2^ prior to cisplatin therapy, and (4) were previously cisplatin-naïve. Patients without cisplatin administration data, non-genotyped patients, and patients of non-European ancestry were excluded from further analyses. Study procedures were approved by the Review Ethics Board of the University Health Network, Toronto, Canada (CAPCR06-639, CAPCR07-0521) and implemented in accordance with the Declaration of Helsinki (64th WMA General Assembly, Fortaleza, Brazil, October 2013). All patients provided the informed written consent.

#### 2.1.2. Validation Cohort

Patients diagnosed with non-small-cell lung cancer (NSCLC) included in the PGxLUNG study were identified as an independent cohort for the purpose of validating the association between any identified variant and nephrotoxicity [21]. Patients of the PGxLUNG study were recruited from one academic hospital (University Medical Center Utrecht), two teaching hospitals (St. Antonius Hospital Nieuwegein/Utrecht, Meander Medical Center Amersfoort) and three general hospitals (Diakonessenhuis Utrecht/Zeist, Groene Hart Ziekenhuis Gouda, Ziekenhuis Rivierenland Tiel), all in the Netherlands, between February 2016 and August 2019. The inclusion criteria for this multicenter prospective follow-up study were as follows: (1) ≥18 years of age, (2) had radiologically confirmed NSCLC (stage II-IV), (3) planned or initiated first-line treatment with platinum-based (cisplatin or carboplatin) chemotherapy or chemoradiotherapy (according to the contemporary ESMO Clinical Practice Guidelines), and (4) were previously platinum-based chemotherapy-naïve. For the analyses as part of this study, patients who did not receive cisplatin and patients of non-European ancestry were excluded. Study procedures were approved by the accredited Medical Research Ethics Committee in Nieuwegein (MEC-U, number R15.056) and implemented in accordance with the Declaration of Helsinki (64th WMA General Assembly, Fortaleza, Brazil, October 2013). All patients provided the informed written consent. Because the inclusion/exclusion and treatments were not identical to the discovery cohort, we have termed this a validation (and not replication) cohort.

### 2.2. Clinical Data Collection

Information on age, sex, weight, height, body surface area (BSA), type of cancer, baseline albumin, concomitant therapy, comorbidities, cisplatin administration (timing and dose) and serum creatinine (SCr) was extracted from the hospitals’ electronic medical record systems. Cisplatin dosage (mg/m^2^) was acquired by dividing the actual cisplatin dose administered (mg) by the BSA.

### 2.3. Cisplatin-Induced Nephrotoxicity Phenotype

Cisplatin-induced nephrotoxicity was defined using two phenotype definitions: (1) the SCr-based CTCAE 4.03 [22] definition of “acute kidney injury” (AKI-CTCAE) as a categorical variable (grade 1 [creatinine level increase of >0.3 mg/dL (≈26 µmol/L); creatinine 1.5 −2.0× above baseline] or higher was defined as nephrotoxicity) and (2) difference between baseline and lowest eGFR (delta) during the follow-up period as a continuous variable. The eGFR was calculated using the Chronic Kidney Disease Epidemiology Collaboration formula (CKD-EPI) as per the Kidney Disease: Improving Global Outcomes (KDIGO) recommendation [23]. Baseline values were defined as the SCr and eGFR measurements taken closest to the first cisplatin administration (within 30 days before the first cisplatin administration).

The follow-up period for the assessment of nephrotoxicity in the discovery and validation cohort was 90 and 21 days after the last cisplatin dose, respectively. Given such a range in kidney function follow up period, AKI-CTCAE can also be defined as acute kidney disease/disorder as per KDIGO Clinical Practice Guideline for Acute Kidney Injury [24]. The follow-up period for the validation cohort was shorter to avoid treatment bias, since some patients in the validation cohort, but not in the discovery cohort, were allowed to switch to carboplatin during therapy, typically 21 days after the last cisplatin dose. In contrast, this switch was not allowed in the patients of the discovery dataset, where we could capture a longer follow-up period of 90 days.

### 2.4. Genotyping and Imputation

DNA was extracted from peripheral blood. Four chips were used for genotyping: the Consortium-OncoArray 500K and OncoArray 500K (Illumina, San Diego, CA, USA) at the Center for Inherited Disease Research (CIDR; Johns Hopkins, Baltimore, MD, USA) for head and neck cancer patients, the Human Omni 1M Quad Beadchip at the US National Cancer Institute (Bethesda, MD, USA) for esophageal cancer patients and the Infinium Global Screening Array-24 Kit (Illumina, San Diego, CA, USA) at Life and Brain (Bonn, Germany) for NSCLC patients. Different genotyping chips were used because this study consists of several independent cohorts that were merged into a discovery and a validation cohort. Sample quality control (QC) was performed for each chip with the following criteria: sample call rate >98%, heterozygosity ±3 SD from the sample’s heterozygosity rate mean, and pi-hat <0.2 to eliminate cryptic relatedness. Genetic ethnicity was analyzed using the multidimensional scaling (MDS) approach based on Human Genome 1K data. We opted to only analyze patients with genetically estimated European ancestry to avoid false-positive genetic association due to inflated test statistics from population stratification. Such results may occur when disease prevalence and allelic frequency differences are correlated within or between study cohorts. The following criteria were used for SNPs QC: SNP call rate > 98%, minor allele frequency (MAF) >0.05, Hardy–Weinberg equilibrium *p* > 10^−6^ (in patients without nephrotoxicity for the AKI-CTCAE phenotype and for the eGFR phenotype) and *p* > 10^−10^ (in patients with nephrotoxicity for the AKI-CTCAE phenotype). Imputation using these QC-passed SNPs was conducted on the University of Michigan Imputation Server [25] using the Minimac4 1.2.1, 1000G Phase 3 v5 reference panel, GRCh37/hg19 array build and Eagle v2.4 phasing. Those SNPs with imputation quality (Rsq) >0.8 and MAF > 0.05 were retained for association analysis. QC was performed using pLINK v.1.9 and 2 [26,27].

### 2.5. Statistical Analysis

#### 2.5.1. Genome-Wide Approach: Discovery Cohort

The sample size needed for the discovery cohort was calculated using GAS Power Calculator [28], assuming an additive model, type I error rate of 5 × 10^−8^, MAF of 20%, cisplatin-induced nephrotoxicity prevalence of 30% and genotype relative risk of 2.0. A minimum of 680 subjects was required to achieve 80% power.

The GWAS assumed additive SNP effects for the AKI-CTCAE phenotype and linear additive effects for the eGFR phenotype. The GWAS was conducted on imputed SNPs and adjusted for 10 genetic MDS components as well as baseline eGFR, sex, age at cisplatin initiation, cumulative dose of cisplatin, cardiovascular disease status, diabetes mellitus status, and chronic NSAID usage. Logistic regression and multiple linear regression analysis were conducted to evaluate the association between genetic variants and the AKI-CTCAE (dichotomous categorical outcome) and eGFR phenotypes (continuous outcome), respectively. Association analysis was performed using pLINK 1.9 [26,27]. Multiple cohort analyses were conducted by combining GWAS results from each genotyping chip in a meta-analysis using the inverse variance method with fixed effect model performed by METAL [29] to overcome issues that might arise from including different genotyping platforms and to increase the power of this study. The Manhattan plot and the Q–Q plot of the GWAS meta-analysis results were visualized using R version 3.4 (http://www.R-project.org/, accessed on 20 February 2021). The genome-wide significance association and suggestive association were set at *p* ≤ 5 × 10^−8^ and *p* ≤ 10^−5^, respectively.

#### 2.5.2. Candidate Gene Approach: Validation Cohort

SNPs meeting at least the suggestive association threshold (*p* ≤ 10^−5^) for each phenotype in the discovery cohort were assessed in the validation cohort. The strength of the association between genotypes and nephrotoxicity phenotypes were evaluated with regression analysis and expressed as odds ratios and β with a 95% confidence interval (CI) for the AKI-CTCAE phenotype and eGFR phenotype, respectively. Association analysis was conducted on imputed SNPs and was adjusted for 10 genetic MDS components as well as sex, age at cisplatin initiation, cumulative dose of cisplatin and Charlson Comorbidity Index [30] (including diabetes mellitus and cardiovascular disease status). The False Discovery Rate (FDR) was used for correction in multiple testing based on the Benjamini–Hochberg procedure available in pLINK [31]. Association analysis was performed using pLINK 1.9 [27], and significant association was set at adjusted *p* < 0.05. The sample size needed for the validation cohort was calculated using GPower [32] based on 80% power, 5% alpha and the results of our discovery dataset (i.e., effect sizes and allele frequency). The minimum sample sizes for AKI-CTCAE and eGFR outcomes were 141 and 153 patients, respectively.

#### 2.5.3. Sensitivity Analysis in the Discovery Cohort

A sensitivity analysis was carried out in the discovery cohort subjects in which the Charlson Comorbidity Index data were available. The GWAS was conducted in the same manner as the primary association analysis except the Charlson Comorbidity Index was incorporated into the model, instead of the specific variables of cardiovascular disease and diabetes mellitus status.

#### 2.5.4. Association of Previously Investigated SNPs Based on the Systematic Review

The relationships between known genetic variants identified in our previously published systematic review [13] and cisplatin-induced nephrotoxicity were also evaluated in the same manner with both discovery and validation cohort analysis.

#### 2.5.5. Population Impact Measures

The potential impact of pharmacogenetic testing, in terms of preventing one patient from having an adverse event, can be expressed as the number needed to genotype (NNG). Furthermore, the number needed to treat (NNT) can be calculated as the number of patients who need an intervention to prevent one patient from having an adverse event, with patients being those who carry the genetic variant indicating the need for alternative treatment. The NNG and NNT on the SNP with strongest evidence were determined using the combined dataset (discovery and validation cohort) to estimate the efficiency of genotyping and treatment modification based on the formula described by Tonk et al. [33].

## 3. Results

### 3.1. Population Characteristics of Discovery and Validation Cohorts

**Figure 1:**
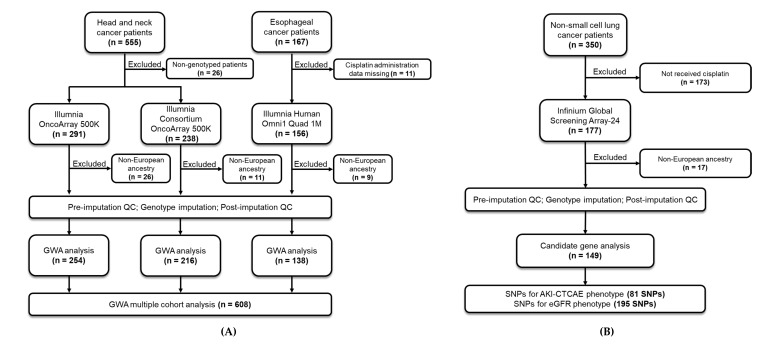
Flowchart of this study. (**A**) Discovery cohort (*n* = 608). The discovery cohort consisted of head and neck cancer patients (*n* = 555) and esophageal cancer patients (*n* = 167). Three arrays were used for genotyping. After pre-imputation QC, imputation and post-imputation QC, data of 608 patients in total were included for analysis. (**B**) Validation cohort (*n* = 149). The validation cohort consisted of non-small-cell lung cancer patients (*n* = 350). After pre-imputation QC, imputation, post-imputation QC and exclusion for chemotherapeutic therapy, 149 patients in total were included for analysis. Abbreviations: AKI-CTCAE (Common Terminology Criteria for Adverse Events v4.03 for acute kidney injury), GWA (genome-wide association), SNPs (single-nucleotide polymorphisms), and QC (quality control).

The study flow chart is shown in Figure 1A (discovery cohort) and Figure 1B (validation cohort). After performing pre- and post-imputation QC and through the MDS approach, data from 608 and 149 patients of European genetic ancestry were available for the discovery cohort and validation cohort, respectively (Figure S1).

The demographic and clinical characteristics of the cohorts are shown in Table 1, while the clinical characteristics categorized by type of cancer (discovery cohort only) are available in the supplement (Table S1). The majority of patients in the discovery cohort were diagnosed with head and neck cancer (470 patients, 77.3%).

Within the discovery cohort, no statistically significant differences were found in gender and percentage of patients with diabetes mellitus between head and neck and esophageal cancer patients. However, mean ± SD age at cisplatin initiation was higher in esophageal cancer patients compared to head and neck cancer patients (59.8 ± 9.6 vs. 57.4 ± 7.3 years). In contrast, the percentage of patients with cardiovascular disease, chronic NSAID users, and treated with radiotherapy were higher in head and neck cancer patients (28.1% vs. 17.4%; 8.3% vs. 2.2%; 98.3% vs. 52.2%, respectively). Among the 509 subjects where data were available to calculate the Charlson Comorbidity Index score, there were no statistically significant differences in Charlson Comorbidity Index score between the head and neck and esophageal cancer patient subgroups (see Table S1). Albumin and eGFR baseline were statistically (but not clinically relevant) significantly higher in head and neck cancer patients (median: 42 vs. 41 mmol/L; 94.3 vs. 92.2 mL/min/1.73 m^2^, respectively).

Compared to the discovery cohort, patients in the validation cohort were statistically significantly older at cisplatin initiation (mean ± SD: 62.8 ± 9.4 vs. 57.9 ± 7.9 years), more frequently female (82.2% vs. 47.7%), had more comorbidities (Charlson Comorbidity Index score ≥ 6: 23.4% vs. 11.0%) and were less often treated with concurrent radiotherapy (58.4% vs. 87.8%). The baseline albumin and eGFR in the discovery cohort was statistically (but not clinically) significantly higher than in the validation cohort (median: 42 vs. 39 mmol/L; 94 vs. 90 mL/min/1.73 m^2^, respectively; Table 1)

### 3.2. Cisplatin-Induced Nephrotoxicity in the Discovery and Validation Cohorts

In the discovery cohort, 93 patients (15.3%) developed grade 1 or higher AKI-CTCAE during cisplatin therapy (Table 2). Data on treatment characteristics and distribution of outcomes within the discovery cohort are shown in Table S2. In both head and neck cancer and esophageal cancer subgroups, subjects with cardiovascular disease, diabetes mellitus, and those who in chronic use of NSAIDs more frequently developed nephrotoxicity (Table S1). The head and neck cancer subgroup received cisplatin as a single agent with a higher cumulative dose of cisplatin (median: 198.2 vs. 173.8 mg/m^2^) and a higher percentage of radiotherapy-treated subjects (98.3% vs. 52.2%). However, the incidence of nephrotoxicity between the two types of cancer was similar (14.9% vs. 16.7%). The percentage of patients with more comorbidities, chronic NSAID use or who had received concurrent administration of other antineoplastics, was higher in patients who developed nephrotoxicity. No statistically significant differences in age at cisplatin initiation or albumin baseline were found between the group of patients with and without nephrotoxicity, both in head and neck and esophageal cancer patients (Table S3).

As shown in Table 2, patients in the validation cohort more frequently developed grade 1 or higher AKI-CTCAE compared to discovery cohort patients (26.8% vs. 15.3%). In both the discovery and validation cohort patients, most of the AKI-CTCAE occurred as grade 1 (11.7% and 22.1%, respectively). Validation cohort patients received a non-significantly higher cumulative dose of cisplatin (median: 224.5 vs. 196.7 mg/m^2^). Validation cohort patients tended to receive a greater number of chemotherapy cycles than patients in the discovery cohort (median: 3 vs. 2 cycles). The reduction in the eGFR was statistically (but not clinically relevant) greater in the validation cohort (median: 11 vs. 7 mL/min/1.73 m^2^) while the median reduction in eGFR between discovery and validation cohort in patients with and without nephrotoxicity was not statistically different.

### 3.3. Association Analysis in the Discovery Cohort

**Figure 2:**
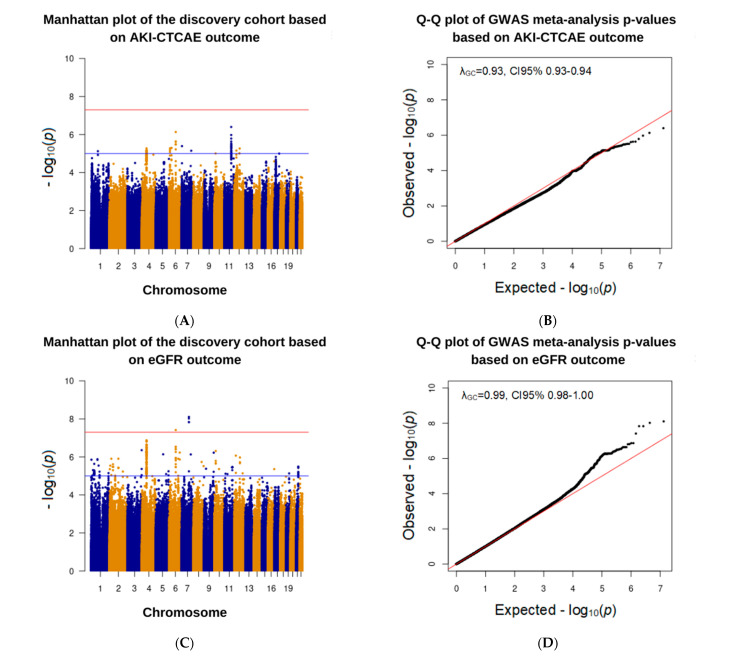
Genome-wide meta-analysis results of cisplatin-induced nephrotoxicity using AKI-CTCAE and eGFR phenotypes. (**A**) Manhattan plot showing logistic regression results using the AKI-CTCAE phenotype; −log10 *p*-values are plotted against the respective chromosomal position of each SNP. (**B**) A quantile–quantile (Q–Q) plot showing the distribution of *p*-values in the GWAS using the AKI-CTCAE phenotype. (**C**) Manhattan plot showing logistic regression results using the eGFR phenotype. (**D**) Q–Q plot showing the distribution of *p*-values in the GWAS using the eGFR phenotype.

After QC processing and initial association analysis, more than 6.5 million SNPs were included in the GWAS meta-analysis of the discovery cohort. The Manhattan plot and Q–Q plot of the analysis can be found in Figure 2A,B. No genomic inflation was observed in the GWAS for the AKI-CTCAE phenotypes as none of the tested SNPs surpassed the genome-wide significance threshold (*p* ≤ 5 × 10^−8^). However, 81 SNPs exceeded the suggestive association *p*-value (*p* ≤ 10^−5^) with most of the signals in SNPs at chromosomes 4, 6, and 11. Details of the top 20 SNPs associated with grade 1 or higher AKI-CTCAE can be found in Table S4.

The Manhattan plot and Q–Q plot of the analysis based on eGFR outcome can be found in Figure 2C,D. Four intronic SNP variants and one variant sitting outside of a known gene that exceeded the genome-wide significance threshold were identified (see Table S5): two SNPs were associated with lower risk for eGFR reduction, *ARPC1A* rs199659233 and rs556958738 (β = 28.7, 95% CI 18.7–38.6, *p* = 1.5 × 10^−8^) and three SNPs were associated with higher risk for eGFR reduction, *TMEM225B* rs17161766 (β = −28.9, 95% CI −38.8–−19.1, *p* = 7.8 × 10^−9^), chr7:98951080 (β = −27.2, 95% CI −36.5–−17.9, *p* = 9.5 × 10^−9^), and *BACH2* rs4388268 (β = −8.4, 95% CI −11.4–−5.4, *p* = 3.9 × 10^−8^). 190 SNPs met suggestive association *p*-value threshold. Of these 195 SNPs, 11 intron variants SNPs are located on chromosome 7, except for rs4388268, which is located on chromosome 6. Of the five SNPs with genome-wide significance, only *BACH2* rs4388268 was consistently surpassed post-imputation QC in three genotyping arrays of the discovery cohort. The remaining four SNPs surpassed the QC in only one of the three datasets. In addition, *BACH2* rs4388268 was consistently associated with a decreased eGFR in the discovery cohort with genome-wide significant association (β = −8.4, 95% CI −11.4–−5.4, *p* = 3.9 × 10^−8^) and with higher risk of the AKI-CTCAE with suggestive association (OR = 3.9, 95% CI 2.3–6.7, *p* = 7.4 × 10^−7^) (Table 3).

The sensitivity analysis in 509 subjects with Charlson Comorbidity Index data confirmed consistent direction of association and similar effect sizes of *BACH2* rs4388268 with previous analysis with regard to cisplatin-induced nephrotoxicity. The variant was consistently associated with a decreased eGFR (β = −8.1, 95% CI −11.4–−4.8, *p* = 1.4 × 10^−6^) and with higher risk of the AKI-CTCAE (OR = 3.6, 95% CI 1.7–5.4, *p* = 3.8 × 10^−5^) (Table S6, Figure S2).

### 3.4. Association Analysis in the Validation Cohort Based on GWAS Results

Following analysis of the discovery cohort, SNPs surpassing the suggestive association threshold (81 SNPs for AKI-CTCAE and 195 SNPs for eGFR outcome in which 32 SNPs were overlapped) were further tested in the validation cohort. Although no statistically significant association was validated, the association of *BACH2* rs4388268 was associated in the same direction as in the discovery cohort for both the AKI-CTCAE (OR = 1.7, 95% CI 0.8–3.5) and eGFR outcomes (β = −1.5, 95% CI −5.3–2.4; Table 4).

### 3.5. Association of Previously Investigated SNPs with Cisplatin-Induced Nephrotoxicity Based on the Systematic Review

A candidate gene approach was also used to study five SNPs identified from our previous systematic review [13]: *ERCC1* rs11615, *ERCC1* rs3212986, *ERCC2* rs13181, *ERCC2* rs1799793 and *SLC22A2* rs316019. However, in the discovery cohort, no significant or suggestive associations were found between these SNPs and either renal toxicity outcome.

In the validation cohort, allele C *ERCC1* rs3212986 was associated with eGFR reduction (β = −4.4, 95% CI −8.1–−0.7). However, the association was no longer statistically significant after multiple-testing adjustment (Table 4).

### 3.6. BACH2 rs4388268 and Risk of Nephrotoxicity

In the discovery cohort, *BACH2* rs4388268 was the SNP most consistently associated, with increased risk of cisplatin-induced nephrotoxicity in both outcomes and across the genotyping platforms, and it met genome-wide significance for the eGFR outcome and suggestive association for AKI-CTCAE. In our validation cohort, this variant was also consistently associated in the same direction for both AKI-CTCAE and eGFR phenotypes although the results were not statistically significant. Closer examination of this variants in both discovery and validation cohorts, revealed that patients with an additional copy of the A allele at rs4388268 were at higher risk for cisplatin-associated nephrotoxicity defined as grade 1 or higher AKI-CTCAE (Figure S3). In the discovery cohort, the incidence of grade 1 or higher AKI-CTCAE was 10.6% for patient with a GG genotype, while the incidence was 24.7% for patients with an AG genotype 36.4% for AA genotype. In the validation cohort, the incidence rates in GG, AG and AA genotype were 24%, 30.4% and 50%, respectively.

In the discovery cohort, an additional copy of the A allele also increased the median eGFR reduction from 6.2 to 9.6 to 13.3 mL/min/1.73 m^2^ for GG homozygotes, AG heterozygotes and AA homozygotes, respectively (Figure S4). A similar trend in eGFR reduction was not observed in the validation cohort. An additional copy of the A allele reduced the median eGFR reduction from 10 to 9 mL/min/1.73 m^2^ for GG and AG heterozygotes, respectively. The eGFR reduction then increased to 13.5 mL/min/1.73 m^2^ for AA homozygotes in the validation cohort. However, the overall trend in the combined dataset still showed continuous reduction (Figure S4) with median eGFR reduction 6.6 for GG, 9.6 for AG and 13.3 mL/min/1.73 m^2^ for AA genotype. A carrier of allele A may experience a reduction in eGFR up to 66 mL/min/1.73 m^2^. The median eGFR reductions for each rs4388268 genotype in overall, discovery, and validation cohorts are available in Table S7.

The NNG and NNT for rs4388268 in the discovery cohort were 44 and 8, respectively while in the validation cohort they were 36 and 7, respectively (Supplementary S1). When both datasets were combined, the NNG and NNT were 42 and 8, respectively (Supplementary S1).

## 4. Discussion

### 4.1. Main Findings

To our knowledge, this is the first GWAS with a validation study in an independent cohort exploring the association between genetic variants and cisplatin-induced nephrotoxicity in cancer patients.rs4388268, an intron variant SNP in the *BACH2* gene, warrants further investigation due to its consistent association with increased risk of cisplatin-induced nephrotoxicity in both AKI-CTCAE and eGFR outcomes and in both discovery and validation cohorts of European ancestry patients. In addition, from five SNPs identified from systematic review, only *ERCC1* rs3212986 was associated with a higher risk of cisplatin-induced nephrotoxicity in the validation cohort of NSCLC patients.

*BACH2* rs4388268 is a common intron variant located in chromosome 6, not only in the European population (MAF = 0.23) but also in the global population (MAF = 0.29) [34]. The frequency of homozygous AA carriers is relatively high, although the European population tends to have a lower frequency than the global population (0.058 vs. 0.103) [35]. Expression quantitative trait loci (eQTLs) data were checked to examine if direct association between genetic variation markers and gene expression levels existed. However, no significant eQTLs were found for this SNP in all tissue types available at Genotype-Tissue Expression project portal (GTEx), meaning that the alternative allele of rs4388268 has no statistically significant effect on any tissue-specific gene expression levels compared to the reference allele [36]. In addition, its low RegulomeDB score of 5 suggests that limited data are available (only transcription factor (TF) binding or Dnase peak available) [37]. The scoring scheme of RegulomeDB ranging from 1 to 7 and refers to the available datatypes for a single coordinate. The highest level of evidence (score 1a) reached when the SNP has the following data: eQTL, TF binding, matched TF motif, matched DNase Footprint and DNase peak [37]. The *BACH2* gene regulates B cell differentiation and function and is therefore biologically relevant for autoimmune disease pathogenesis. Variants in this gene have been previously associated with an increased risk of autoimmune diseases such as Addison’s disease [38], rheumatoid arthritis [39], inflammatory bowel disease [40] and hyperthyroidism [41,42]. One study found BACH2, a transcription regulator protein, to be highly expressed in bone marrow and lymphoid tissue but moderately expressed in kidney tubule [43]. Another study using mouse fibroblast cell line NIH3T3 reported *Bach2* as a rapid and highly sensitive reporter of DNA damage and demonstrated that *Bach2* overexpression is harmful to cell survival while silencing stimulates cell growth and shows protection from acute oxidative stress [44]. A recently published study [45] also showed that aged *Bach2^ΔCD4^* mice displayed prominent IgG deposits in kidney glomeruli suggesting an autoimmunity process. Since cisplatin is mainly excreted through the kidneys, variants in *BACH2* might play a role in the pathogenesis of cisplatin-induced nephrotoxicity, though through which mechanism (cell proliferation, DNA damage, or autoimmunity) is unclear and warrants further investigation.

AKI-CTCAE is commonly used in clinical settings and previous candidate gene studies to measure kidney function. In addition to assessing AKI-CTCAE, this study also evaluated the change in eGFR as a continuous outcome, since age, sex, race, and body weight affect SCr concentration independently from GFR [23]. Genome-wide significance signals were identified for the eGFR outcome, while the CTCAE-AKI outcome only showed SNPs with suggestive association. This is understandable since categorizing a continuous outcome results in loss of information; thus, the statistical power to detect a relation between the SNPs and kidney function was reduced [46]. Moreover, we corrected the association with known risk factors of cisplatin-induced nephrotoxicity. Approximately 15% of the patients in the discovery cohort and 25% of the patients in the validation cohort developed AKI-CTCAE, mainly grade 1, which is lower than the average percentage reported previously [7]. This might be due to effective mitigation strategies such as intravenous fluid repletion, magnesium supplementation and/or the mannitol administration protocol implemented in patient cohorts receiving high-dose cisplatin.

In addition, we could not validate the findings of the previously published candidate gene study on *ERCC1* (rs11615 and rs3212986), *ERCC2* (rs13181 and rs1799793), and *SLC22A2* (rs316019) in our head and neck and esophageal cancer discovery cohort. However, our NSCLC validation cohort showed that rs3212986, a 3 prime UTR variant of *ERCC1*, was associated with a higher risk of cisplatin-induced nephrotoxicity, a result that was in line with previous studies [47,48]. Polymorphisms in *ERCC1* might exhibit the renal tubular damage caused by cisplatin through altered DNA repair mechanisms in the kidney. eQTL data in renal tubular tissue were available to confirm the impact of this SNP on *ERCC1* gene expression [49]. As for other SNPs, inconsistencies in the direction of association were discovered when comparing the association in the validation cohort with previous studies [13]. One possible explanation for the lack of association for these SNPs is population stratification. However, the SNPs of interest, especially five SNPs identified from our systematic review, were also studied in European ancestry subjects and still showed association, except for rs316019 which also studied in East Asian populations [13]. In fact, the allele frequency of rs316019 is comparable between European and East Asian population (0.10 vs. 0.11%) [34]. Other possible explanations for this lack of association are lack of study power, heterogeneity in outcome (i.e., differences in outcome definition and/or differences in cut-off value to be considered as a case) and differences in cancer type which eventually lead to differences in cisplatin-based regimen.

A recently published GWAS reported that rs1377817, a SNP intronic to *MYH14*, was associated with a high residual serum platinum level and possibly correlated to the development of several cisplatin-related toxicities such as tinnitus and Raynaud’s phenomenon [50]. Our previously published candidate gene study also found that addition of allele A at *SLC22A2* rs316019 was associated with an increased risk of grade 1 or higher AKI-CTCAE [51]. However, in the present study significant associations were not found between those SNPs and either of our renal toxicity outcomes, although non-significant associations were in the same direction.

Compared to the discovery cohort, the follow-up period of the validation cohort was shorter. The reason for this is the fact that one-third of the NSCLC patients in the validation cohort were switched to carboplatin-based chemotherapy during treatment and effectively started 21 days after the last administration of cisplatin. These patients were switched to carboplatin for different reasons, but mostly due to cisplatin-induced toxicity. Meanwhile, only 2% of the subjects switched to carboplatin in the discovery cohort. To avoid treatment bias, the follow-up period of 21 days after the last administration of cisplatin was selected instead of 90 days as in the discovery cohort. Since the time-to-AKI is expected to be less than 21 days after cisplatin administration [52], this is arguably an acceptable follow-up duration, although different from the follow-up duration of the discovery cohort.

Differences in clinical characteristics between the discovery and the validation cohort, such as age at cisplatin initiation and number of comorbidities, potentially caused a higher incidence of cisplatin nephrotoxicity in the validation cohort. Such differences may also explain the non-significant contribution of genetic factors on cisplatin nephrotoxicity in the validation cohort. The clinical characteristics could be seen as effect modifiers since such factors were unlikely to confound the association between SNPs and cisplatin nephrotoxicity. Despite the differences in type of cancer (which led to different clinical characteristics), such approach could open possibility to gain more knowledge on the clinical relevance of genetic predisposition on cisplatin nephrotoxicity in different patient populations.

### 4.2. Potential Clinical Relevance

In clinical practice, occurrence of AKI-CTCAE grade 1 or higher will frequently result into clinical interventions such as delaying chemotherapy, cisplatin dose reduction up to 75% or treatment switch (e.g., to carboplatin). Our results indicate the possible involvement of genetic variants in platinum renal disposition. Genetic polymorphisms in *BACH2* were associated with higher risk of cisplatin-induced nephrotoxicity among European ancestry patients. This finding, together with proven clinical risk factors, may facilitate the identification of individuals at high risk of nephrotoxicity despite adequate volume status, magnesium supplementation and mannitol in high-dose cisplatin.

Based on the NNG and NNT in our combined cohort of patients of European ancestry, for every 42 cisplatin-candidate patients who are genotyped, 8 patients will carry a minor allele A of rs4388268. What we demonstrated was that carrying the minor allele A may contribute to susceptibility to nephrotoxicity and interindividual differences in clinical management. Thus, an intervention such as the need to delay, reduce or switch treatment may be considered for almost 20% of patients who are cisplatin candidates, which could have a significant impact on clinical care.

### 4.3. Strengths and Limitations

The present study has several strengths. Firstly, to our knowledge, this is the first GWAS study to investigate the association between genetic variants and cisplatin-induced nephrotoxicity. Secondly, we were able to perform a validation (but not replication) study in an independent cohort. We recognize that both validation and replication will eventually become essential to confirm associations discovered via GWASs, to rule out associations due to bias, to improve effect estimation and to improve understanding of the biological underpinnings [53]. This is a first step towards these goals. Thirdly, the variables collected in our discovery cohort and validation cohort were based on real-world data. Therefore, the results of this study reflect the actual clinical setting, which strengthens the possibility of extrapolating our findings. Finally, although not statistically significant, the effect sizes of the validation study were in same direction as in the discovery cohort, despite the differences in clinical characteristics, type of malignancies, chemotherapy regimen and period of follow-up, suggesting a consistent association between particular genetic variants and cisplatin-induced nephrotoxicity.

The present analysis has some limitations, which illustrate the difficulties of performing such pharmacogenomic studies. First, this study had a relatively small occurrence of grade 2 or higher AKI-CTCAE. Thus, although SNPs were identified that reached genome-wide significance across mild nephrotoxicity, suggesting a strong genetic signal in the development of cisplatin-induced nephrotoxicity, further analysis in this more severe nephrotoxicity group was not feasible. In addition, we had anticipated a higher rate of nephrotoxicity (based on data from older studies) that never materialized. Consequently, the study power was lower than expected. Second, our outcomes relied on the widely used SCr-based nephrotoxicity grading. Serum creatinine is not an ideal biomarker for drug-induced kidney injury because it is influenced by renal and non-renal factors independent of kidney function [54]. In addition, creatinine (to a small extent) competes with cisplatin for excretion as both are substrates of the organic cation transporter 2 (OCT2) [55]. Third, dehydration and chemotherapy-induced nausea and vomiting cases were difficult to detect due to the retrospective nature of the discovery cohort. As for the validation cohort, such data were only partially recorded. Information regarding hydration protocols or other prophylaxis against nephrotoxicity was not available for both cohorts as well. Finally, our study focused on populations of European descent. Thus, further independent investigation should be conducted to assess if the results are transferrable to a more diverse population.

This study highlights both the benefits and limitations of using real-world observational data in pharmacogenomic studies: (i) we utilized pragmatic if imperfect surrogate markers of outcome (e.g., SCr-based changes) that may lead to variability in results; (ii) heterogeneity of populations could lead to heterogeneous results, including variability in eligibility criteria (study population), underlying clinical risks of the drug toxicity (e.g., differences across study cohorts in terms of age, and sex), and treatment regimens (doses and frequency of administration, concurrent drugs and/or radiation); and (iii) the need to validate and replicate results. In our study, we have restricted the focus on validation of the genetic associations but not true replication of results. Despite all of these issues, we were still able to identify a previously unknown variant in *BACH2* as a putative marker of nephrotoxicity.

### 4.4. Future Research

Future studies should focus on functional validation of the *BACH2* role in cisplatin-induced nephrotoxicity, for example through experimental studies in knock-out mice and/or in vitro studies allowing unraveling the molecular pathway. The current issues with using SCr as the basis of nephrotoxicity is a pragmatic approach, but confirmatory studies may require the further development of more sensitive markers of kidney injury. Regardless, if further validated or even replicated in other large datasets of prospective studies with more clinical similarities (e.g., same type of cancers), a clinical study to investigate the potential use of *BACH2* variants in guiding selection of platinum agents (i.e., between cisplatin and carboplatin) to avoid both acute and chronic nephrotoxicity without compromising the platinum’s effectiveness (i.e., radiological response and overall survival) would be a future step. In addition, prospective observational studies that defines nephrotoxicity through highly sensitive and specific urinary biomarkers such as kidney injury molecule-1 (KIM-1), β2-microglobulin (B2M), cystatin C, clusterin, calbindin, neutrophil gelatinase-associated lipocalin (NGAL) and trefoil factor-3 (TFF-3) [54] would enhance understanding of cisplatin-induced nephrotoxicity as showed in a recent pharmacokinetic study [56] and a candidate gene study [57] alongside pragmatic studies such as ours that uses what is currently available in clinical practice.

## 5. Conclusions

The present GWAS and validation study suggest that genetic predisposition could be important in the development of nephrotoxicity among cisplatin users. *BACH2* rs4388268, a common intronic variant, increased the risk of cisplatin-induced nephrotoxicity nearly 4- and 1.7-fold in the discovery and validation cohorts, respectively. These results need further functional and pharmacokinetic/dynamic validation to reveal the mechanistic basis on how the variant may be involved in cisplatin-induced nephrotoxicity. Further replication in an independent cohort is also necessary before this finding can be utilized to personalize cisplatin therapy. In the validation cohort, one of the previously studied candidate SNPs, *ERCC1* rs3212986, was associated with eGFR reduction although the association was no longer statistically significant after multiple-testing adjustment. Nevertheless, genetic predisposition of *BACH2* could be important in the development of cisplatin-induced nephrotoxicity and providing opportunities for mechanistic understanding, potential individualized platinum selection and preventive strategies.

## Figures and Tables

**Table 1:** Demographic and clinical characteristics.

Characteristics	Discovery Cohort (*n* = 608)	Validation Cohort (*n* = 149)	*p*-Value
Age at cisplatin initiation in years, mean ± SD	57.9 ± 7.9	62.8 ± 9.4	<0.01 *
Male, *n* (%)	500 (82.2)	71 (47.7)	<0.01 *
Cardiovascular disease, *n* (%)	156 (25.7)	NA	NA
Diabetes mellitus, *n* (%)	44 (7.2)	NA	NA
Charlson Comorbidity Index ^#^, *n* (%)			
2–3	206 (40.5)	71 (47.7)	<0.01 *
4–5	247 (48.5)	43 (28.9)	
≥6	56 (11.0)	35 (23.4)	
Missing data	99	0	
Chronic NSAID users, *n* (%)	42 (6.9)	NA	NA
Concurrent administration of other antineoplastics, *n* (%)	138 (22.7)	149 (100)	<0.01 *
Received radiotherapy, *n* (%)	534 (87.8)	87 (58.4)	<0.01 *
Albumin baseline, median mmol/L (IQR)	42 (40–44)	39.0 (33.0–42.0)	<0.01 *
Baseline eGFR, median mL/min/1.73 m^2^ (IQR)	94.0 (83.4–101.4)	90.0 (80.0–90.0)	<0.01 *

NA, information not available; NSAID, non-steroidal anti-inflammatory drug; SD, standard deviation; eGFR, estimated glomerular filtration rate; IQR, interquartile range. ^#^ Charlson Comorbidity Index score provides a simple means to quantify the effect of comorbid illnesses, including cardiovascular diseases, chronic obstructive pulmonary disease, liver disease and diabetes mellitus among others and, accounts for the aggregate effect if multiple concurrent diseases. A higher score indicates more comorbidities. * *p*-value < 0.05 based on independent *t*-test or Mann–Whitney *U* Test (for continuous independent variable) and Fisher’s Exact Test or chi-square (for categorical independent variable).

**Table 2:** Treatment characteristics and distribution of outcomes.

Characteristics	Discovery Cohort (*n* = 608)	Validation Cohort (*n* = 149)	*p*-Value
Cumulative dose of cisplatin, median mg/m^2^ (IQR)	196.7 (173.0–248.0)	224.5 (150.1–274.8)	0.297
Cycles of cisplatin-based chemotherapy, *n* (%)			<0.01 *
1	50 (8.2)	28 (18.8)	
2	313 (51.5)	23 (15.4)	
3	201 (33.1)	55 (36.9)	
≥4	44 (7.2)	43 (28.9)	
AKI-CTCAE, *n* (%) ^#^			<0.01 *
Grade 0 (no nephrotoxicity)	515 (84.7)	109 (73.2)	
Grade 1	71 (11.7)	33 (22.1)	
Grade 2	17 (2.8)	4 (2.7)	
Grade 3	5 (0.8)	3 (2.0)	
Grade 4	0 (0)	0 (0)	
Any grade	93 (15.3)	40 (26.8)	
Reduction in eGFR, median, mL/min/1.73 m^2^ (IQR) ^$^	7.0 (0.6–18.9)	11.0 (1.0–25.5)	<0.01 *
Patients without nephrotoxicity	5.5 (0.0–14.3)	7.0 (0.0–16.0)	0.502
Patients with grade 1 or higher AKI-CTCAE	30.6 (15.3–42.9)	34.5 (25.3–41.5)	0.173

IQR, interquartile range; eGFR, estimated glomerular filtration rate. ^#^ Highest AKI-CTCAE grade between cisplatin initiation and the last day of follow-up. ^$^ Differences between baseline eGFR and eGFR nadir recorded from cisplatin initiation until the last day of follow-up. * *p*-value < 0.05 based on Mann–Whitney *U* Test (for continuous independent variable) and chi-square test (for categorical independent variable).

**Table 3:** Association between *BACH2* rs4388268 and cisplatin-induced nephrotoxicity in the discovery cohort.

Chromosome: Location: Allele ^a^	Functional Consequences	Outcome	Effect Size (95% CI) ^b^	*p*-Value	Direction ^c^
6:90734908:G:A	Intron variant	AKI–CTCAE	3.9 (2.3–6.7)	7.4 × 10^−7^	+ + +
		eGFR reduction	−8.4 (−11.4–−5.4)	3.9 × 10^−8^	− − −

^a^ Chromosome: base pair:Allele1:Allele2; ^b^ OR for AKI-CTCAE phenotype and β for eGFR phenotype; ^c^ Three symbols depict the direction of association in the three datasets included in the discovery cohort. The first symbol is for head and neck cancer genotyped with Illumina OncoArray (*n* = 254), the second symbol is for head and neck cancer genotyped with Illumina Consortium OncoArray (*n* = 216), and the third symbol is for esophageal cancer (*n* = 138). For AKI–CTCAE outcome: (−) protective effect; (+) risk effect. For eGFR reduction outcome: (−) reduced eGFR; (+) increased eGFR.

**Table 4:** Association analysis in the validation cohort.

RsID	Genes	Chromosome: Location: Allele ^a^	*Effect Size* (95% CI) ^b^	Unadjusted *p*-Value	Adjusted *p*-Value	Functional Consequences
**Analysis of SNPs that meet at least the suggestive association threshold in the discovery cohort**						
*AKI-CTCAE phenotype* ^c^						
rs4388268	*BACH2*	6:90734908:G:A	1.7 (0.8–3.5)	0.19	0.70	Intron variant
*eGFR phenotype* ^c^						
rs17161766	*TMEM225B*	7:99177716:G:A	NA	NA	NA	Intron variant
NA	NA	7:98951080:C:CTTAT	NA	NA	NA	NA
rs199659233	*ARPC1A*	7:98959960:T:C	NA	NA	NA	Intron variant
rs556958738	*ARPC1A*	7:98959961:T:C	NA	NA	NA	Intron variant
rs4388268	*BACH2*	6:90734908:G:A	−1.5 (−5.3–2.4)	0.45	0.99	Intron variant
**Analysis of known SNPs from systematic review**						
*AKI-CTCAE phenotype*						
rs316019	*SLC22A2*	6:160670282:A:C	1.2 (0.4–3.6)	0.73	0.82	Missense variant
rs13181	*ERCC2*	19:45854919:T:G	0.6 (0.38–1.1)	0.095	0.24	Stop gained
rs1799793	*ERCC2*	19:45867259:C:T	0.5 (0.3–1.1)	0.075	0.24	Missense variant
rs3212986	*ERCC1*	19:45912736:C:A	0.9 (0.4–1.9)	0.82	0.82	3 prime UTR variant
rs11615	*ERCC1*	19:45923653:A:G	1.4 (0.7–2.5)	0.35	0.59	Synonymous variant
*eGFR phenotype*						
rs316019	*SLC22A2*	6:160670282:A:C	1.9 (−3.4–7.2)	0.49	0.82	Missense variant
rs13181	*ERCC2*	19:45854919:T:G	0.09 (−3.2–3.4)	0.96	0.96	Stop gained
rs1799793	*ERCC2*	19:45867259:C:T	−0.3 (−3.8–3.3)	0.89	0.96	Missense variant
rs3212986	*ERCC1*	19:45912736:C:A	−4.4 (−8.1–−0.7)	0.02	0.10	3 prime UTR variant
rs11615	*ERCC1*	19:45923653:A:G	−1.7 (−4.8–1.5)	0.31	0.77	Synonymous variant

^a^ Chromosome: base pair:Allele1:Allele2; ^b^ OR for AKI-CTCAE phenotype and β for eGFR phenotype; ^c^ No significant association was found based on both AKI-CTCAE and eGFR phenotypes; NA, information not available. SNPs did not pass the quality control.

## Data Availability

Datasets generated and analyzed during this study (patient clinical and genetic data) are not publicly available due to patient privacy protection. Such data are available from the principal investigator for each cohort on reasonable request. For data access requests on the discovery cohort, please contact Geoffrey Liu, Division of Medical Oncology and Hematology, Department of Medicine, Princess Margaret Cancer Centre, University of Toronto, Toronto, ON, Canada, e-mail address: Geoffrey.Liu@uhn.ca. For data access request on the validation cohort (PGxLUNG study), please contact V.H.M. Deneer, Department of Clinical Pharmacy, University Medical Center Utrecht, Utrecht, the Netherlands, e-mail address: v.h.m.deneer@umcutrecht.nl.

## Supplementary Materials

The following are available online at https://www.mdpi.com/article/10.3390/jpm11111233/s1, Table S1: Demographic and clinical characteristics in the discovery cohort: head and neck cancer and esophageal cancer patients; Table S2: Treatment characteristics and distribution of outcomes in the discovery cohort: head and neck cancer vs. esophageal cancer patients; Table S3: Demographic and clinical characteristics of patients without nephrotoxicity and patients with grade 1 or higher AKI-CTCAE, both in discovery and validation cohort; Table S4: Top twenty SNPs from genome-wide meta-analysis of cisplatin-induced AKI-CTCAE in the discovery cohort; Table S5: Top twenty SNPs from genome-wide meta-analysis of cisplatin-induced eGFR reduction in the discovery cohort; Table S6: Association between *BACH2* rs4388268 and cisplatin-induced nephrotoxicity in subjects of discovery cohort with available Charlson Comorbidity Index data (*n* = 509); Table S7: Median of eGFR reduction for each *BACH2* rs4388268 genotype in the overall, discovery, and validation cohort; Figure S1: Multidimensional scaling (MDS) plot of 1KG against the subjects of the discovery cohort (A, B and C) and the validation cohort (D) for each genotyping chip; Figure S2: Genome-wide meta-analysis results of cisplatin-induced nephrotoxicity using AKI-CTCAE and eGFR phenotypes in subjects of discovery cohort with available Charlson Comorbidity Index data (*n* = 509); Figure S3: AKI-CTCAE status for each *BACH2* rs4388268 genotype; Figure S4: eGFR differences (ΔeGFR) for each *BACH2* rs4388268 genotype. Supplementary S1: Calculations number needed to genotype (NNG) and number needed to treat (NNT) on *BACH2* rs4388268 based on formula provided by Tonk, et al. (2017).
