# Supplementary material for: Association between Genetic Variants and Cisplatin-Induced Nephrotoxicity: A Genome-Wide Approach and Validation Study
Source: J Pers Med. 2021 Nov 20;11(11):1233. doi: 10.3390/jpm11111233 (PMC8623115; doi:10.3390/jpm11111233)
Supplement: Supplementary file 1 [file jpm-11-01233-s001.zip › jpm-1447892-supplementary.pdf]

Association between Genetic Variants and Cisplatin-Induced Nephrotoxicity: A Genome-Wide Approach and Validation Study

Supplementary Files

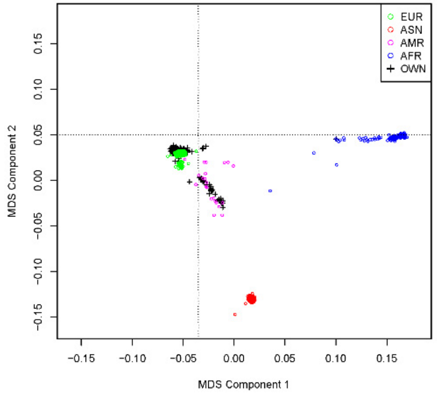

(A)

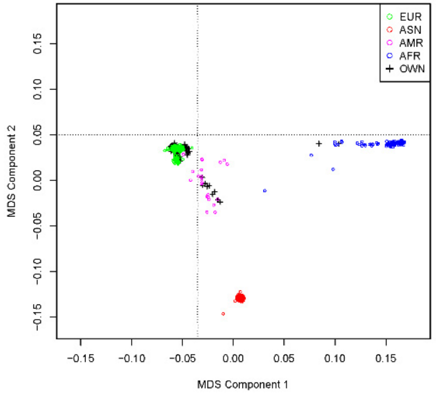

(B)

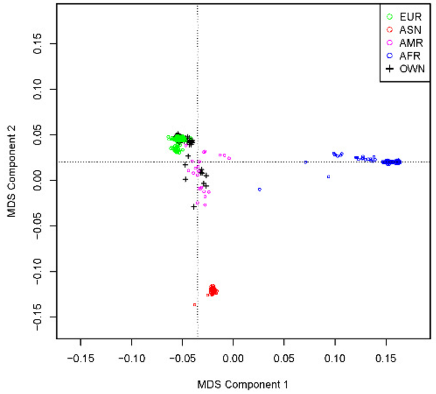

(C)

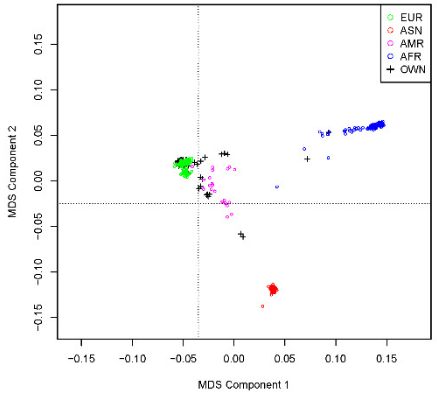

(D)

Figure S1. Multidimensional scaling (MDS) plot of 1KG against the subjects of the discovery cohort (A, B and C) and the validation cohort (D) for each genotyping chip. The black crosses (+ = "OWN") in the upper right part represent the first two MDS components of the individuals in the subjects (the colored symbols represent the 1KG data (O = European; □ = African; X = Ad Mixed American; Δ = Asian). The MDS components representing the European samples (O) are located in the upper left, the African samples (□) are located in the upper right, the Ad Mixed American samples (X) are located in the center, the Asian components (Δ) are located in the lower part.

Table S1. Demographic and clinical characteristics in the discovery cohort: head and neck cancer and esophageal cancer patients

Characteristics	Head and neck cancer patients			Esophageal cancer patients			*P*-value[a]
	Total (*n* = 470)	Nephrotoxicity (grade 1 or higher AKI-CTCAE)		Total (*n* = 138)	Nephrotoxicity (grade 1 or higher AKI-CTCAE)		
		No (*n* = 400)	Yes (*n* = 70)		No (*n* = 115)	Yes (*n* = 23)	
Age at cisplatin initiation in years, mean±SD	57.4±7.3	57.3±7.3	57.0±7.3	59.8±9.6	60.2±9.4	58.0±10.6	< 0.01*
Male, *n* (%)	387 (82.3)	328 (82)	59 (84.3)	113 (81.9)	92 (80)	21 (91.3)	0.90
Cardiovascular disease, *n* (%)	132 (28.1)	106 (26.5)	26 (37.1)	24 (17.4)	15 (13)	9 (39.1)	0.01*
Diabetes mellitus, *n* (%)	35 (7.4)	25 (6.3)	10 (14.3)	9 (6.5)	5 (4.3)	4 (17.4)	0.85
Charlson Comorbidity Index[#], *n* (%)							
2–3	175 (42.2)	156 (44.3)	19 (30.2)	31 (33.0)	28 (36.4)	3 (17.6)	0.23
4–5	197 (47.5)	163 (46.3)	34 (54.0)	50 (53.2)	39 (50.6)	11 (64.7)	
≥ 6	43 (10.4)	33 (9.4)	10 (15.9)	13 (13.8)	10 (13.0)	3 (17.6)	
Missing data	55	48	7	44	38	6	
Chronic NSAID users, *n* (%)	39 (8.3)	32 (8)	7 (10)	3 (2.2)	2 (1.7)	1 (4.3)	0.01*
Concurrent administration of other antineoplastics, *n* (%)	0 (0)	0 (0)	0 (0)	138 (100)	115 (100)	23 (100)	NA
Received radiotherapy, *n* (%)	462 (98.3)	393 (98.3)	69 (98.6)	72 (52.2)	63 (54.8)	9 (39.1)	< 0.01*
Albumin baseline, median mmol/L (IQR)	42 (41-44)	42 (40-44)	43 (41-44)	41 (39-43)	41 (39-43)	41 (39-43)	< 0.01*
Baseline eGFR, median mL/min/1.73 m^2 (IQR)	94.3 (85.2-101.5)	94.6 (84.8-101.5)	93.8 (88.3-101.5)	92.2 (77.4-100.4)	92.2 (79-99.9)	90.6 (68.3-105.7)	0.02*

NA, information not available; NSAID, non-steroidal anti-inflammatory drug; SD, standard deviation; eGFR, estimated glomerular filtration rate; IQR, interquartile range.

[#] Charlson Comorbidity Index score provides a simple means to quantify the effect of comorbid illnesses, including cardiovascular diseases, chronic obstructive pulmonary disease, liver disease and diabetes mellitus among others and, accounts for the aggregate effect if multiple concurrent diseases. A higher score indicates more comorbidities.

[a] *P*-value of comparison head and neck and esophageal cancer patients

* *P*-value < 0.05 based on independent t-test or Mann-Whitney U Test (for continuous independent variable) and Fisher's Exact Test or chi-square (for categorical independent variable)

Table S2. Treatment characteristics and distribution of outcomes in the discovery cohort: head and neck cancer vs. esophageal cancer patients

**Characteristics**	**Head and neck cancer patients (*n* = 470)**	**Esophageal cancer Patients (*n* = 138)**	***P*-value**
Cumulative dose of cisplatin, median mg/m^2 (IQR)	198.2 (179.6-250)	173.8 (140.6-222.8)	< 0.01*
Cycles of cisplatin-based chemotherapy, *n* (%)			< 0.01*
1	35 (7.4)	15 (10.9)	
2	275 (58.5)	38 (27.5)	
3	155 (33)	46 (33.3)	
≥4	5 (1.1)	39 (28.3)	
AKI-CTCAE, *n* (%)[#]			0.61
Grade 0 (no nephrotoxicity)	400 (85.1)	115 (83.3)	
Grade 1	51 (10.9)	20 (14.5)	
Grade 2	14 (3)	3 (2.2)	
Grade 3	5 (1.1)	0 (0)	
Grade 4	0 (0)	(0)	
Any Grade	70 (14.9)	23 (16.7)	
Reduction in eGFR, median, mL/min/1.73 m2 (IQR)[$]	6.6 (0.5-18.6)	8.9 (1.1-19.3)	0.28
Patients without nephrotoxicity	5.1 (0.0-13.6)	6.8 (0.0-16.1)	0.22
Patients with grade 1 or higher AKI-CTCAE	32.1 (19.4-46.6)	25.5 (10.1-38.7)	0.14

IQR, interquartile range; eGFR, estimated glomerular filtration rate.

[#] Highest AKI-CTCAE grade between cisplatin initiation and the last day of follow-up.

[$] Differences between baseline eGFR and lowest eGFR recorded from cisplatin initiation until the last day of follow-up.

* *P*-value < 0.05 based on Mann-Whitney U Test (for continuous independent variable) and chi-square test (for categorical independent variable).

Table S3. Demographic and clinical characteristics of patients without nephrotoxicity and patients with grade 1 or higher AKI-CTCAE, both in discovery and validation cohort

**Characteristics**	**Discovery cohort (*n* = 608)**	**Nephrotoxicity (grade 1 or higher AKI-CTCAE)**		**Validation cohort (*n* = 149)**	**Nephrotoxicity (grade 1 or higher AKI-CTCAE)**		***P*-value[a]**
		**No (*n* = 515)**	**Yes (*n* = 93)**		**No (*n* = 109)**	**Yes (*n* = 40)**	
Age at cisplatin initiation in years, mean±SD	57.9 ± 7.9	57.9 ± 7.9	58.0 ± 8.2	62.8 ± 9.4	62.8 ± 9.6	62.8 ± 9.2	< 0.01*
Male, *n* (%)	500 (82.2)	420 (81.6)	80 (86.0)	71 (47.7)	52 (47.7)	19 (47.5)	< 0.01*
Cardiovascular disease, *n* (%)	156 (25.7)	121 (23.5)	35 (37.6)	NA	NA	NA	NA
Diabetes mellitus, *n* (%)	44 (7.2)	30 (5.8)	14 (15.1)	NA	NA	NA	NA
Charlson Comorbidity Index[#], *n* (%)							< 0.01*
2–3	206 (40.5)	184 (42.9)	22 (27.5)	71 (47.7)	51 (46.8)	20 (50.0)	
4–5	247 (48.5)	202 (47.1)	45 (56.3)	43 (28.9)	30 (27.5)	13 (32.5)	
≥ 6	56 (11.0)	43 (10.0)	13 (16.3)	35 (23.4)	28 (25.7)	7 (17.5)	
Missing data	99	86	13	0	0	0	
Chronic NSAID users, *n* (%)	42 (6.9)	34 (6.6)	8 (8.6)	NA	NA	NA	NA
Concurrent administration of other antineoplastics, *n* (%)	138 (22.7)	115 (22.3)	23 (24.7)	149 (100)	109 (100)	40 (100)	< 0.01*
Received radiotherapy, *n* (%)	534 (87.8)	456 (88.5)	78 (83.9)	87 (58.4)	73 (67.0)	14 (35.0)	< 0.01*
Albumin baseline, median mmol/L (IQR)	42 (40–44)	42 (40–44)	42 (41–44)	39.0 (33.0–42.0)	39.0 (32.0–42.0)	39.0 (34.6–42.0)	< 0.01*
Baseline eGFR, median mL/min/1.73 m^2 (IQR)	94.0 (83.4–101.4)	94.0 (83.1–101.2)	93.7 (86.3–101.7)	90.0 (80.0–90.0)	90.0 (79.5–90.0)	90.0 (81.0–90.0)	< 0.01*

NA = Information not available; NSAID, non-steroidal anti-inflammatory drug; SD, standard deviation; eGFR, estimated glomerular filtration rate; IQR, interquartile range.

[#] Charlson Comorbidity Index score provides a simple means to quantify the effect of comorbid illnesses, including cardiovascular diseases, COPD, liver disease and diabetes mellitus among others and, accounts for the aggregate effect if multiple concurrent diseases. A higher score indicates more comorbidities.

[a] *P*-value of comparison head and neck and esophageal cancer patients.

* *P*-value < 0.05 based on independent t-test or Mann-Whitney U Test (for continuous independent variable) and Fisher's Exact Test or chi-square (for categorical independent variable).

Table S4. Top twenty SNPs from genome-wide meta-analysis of cisplatin-induced AKI-CTCAE in the discovery cohort

rsID	Gene	Chromosome:Location :Allele[a]	OR (95% CI)	P-value	Direction[b]	Heterogeneity I^2	Heterogeneity P-value	Functional Consequences	eQTL from GTEx database	RegulomeDB score
NA	NA	11:94417672:AC:A	3.4 (2.1-5.5)	$5.0x10^{-7}$	+++	53.9	0.11	NA	NA	NA
rs4388268	*BACH2*	6:90734908:G:A	3.9 (2.3-6.7)	$7.4x10^{-7}$	+++	0	0.98	intron variant	no significant data	5
rs72965891	*LOC105369438*	11:94415630:T:C	0.3 (0.2-0.5)	$1.1x10^{-6}$	---	60	0.08	intron variant	no significant data	5
rs11020896	*LOC105369438*	11:94416887:T:C	0.3 (0.2-0.5)	$1.6x10^{-6}$	---	28.7	0.25	intron variant	no significant data	No data
rs16882364	*BACH2*	6:90735491:A:G	0.2 (0.1-0.4)	$2.3x10^{-6}$	---	29.2	0.24	intron variant	no significant data	2b
rs7110345	*LOC105369438*	11:94395048:G:A	3.0 (1.9-4.6)	$2.3x10^{-6}$	+++	77.8	0.01	intron variant	no significant data	No data
rs12664728	*BACH2*	6:90737315:G:A	5.4 (2.7-10.9)	$2.5x10^{-6}$	+++	30.1	0.24	intron variant	no significant data	6
NA	NA	11:94449088:GC:G	3.0 (1.9-4.8)	$3.1x10^{-6}$	+++	38.5	0.20	NA	NA	NA
rs7350489	*AMOTL1; LOC105369438*	11:94452476:C:T	3.0 (1.9-4.8)	$3.1x10^{-6}$	+++	38.5	0.20	intron variant	no significant data	No data
NA	NA	11:94451910:GTTGA:G	3.0 (1.9-4.8)	$3.2x10^{-6}$	+++	38.1	0.20	NA	NA	NA
rs16882357	*BACH2*	6:90731445:T:C	0.2 (0.1-0.4)	$3.3x10^{-6}$	---	25.7	0.26	intron variant	no significant data	5
rs12664550	*BACH2*	6:90732877:T:C	0.2 (0.1-0.4)	$3.3x10^{-6}$	---	25.7	0.26	intron variant	no significant data	6

rs60917421	*LOC105369438*	11:94395777:T:C	0.4 (0.2-0.5)	3.5×10^{-6}	---	78.2	0.01	intron variant	no significant data	2b
rs11020924	*AMOTL1; LOC105369438*	11:94453131:A:G	0.3 (0.2-0.5)	3.9×10^{-6}	---	32.2	0.23	intron variant	no significant data	6
rs2068908	*AMOTL1; LOC105369438*	11:94458154:G:A	3.0 (1.9-4.7)	3.9×10^{-6}	+++	32.2	0.23	intron variant	no significant data	6
rs11020920	*AMOTL1; LOC105369438*	11:94447905:T:C	0.3 (0.2-0.5)	4.0×10^{-6}	---	32.1	0.23	intron variant	no significant data	6
rs4486099	*TNRC18*	7:5373370:A:C	2.9 (1.9-4.6)	4.1×10^{-6}	+++	62.8	0.07	intron variant	no significant data	5
rs10831271	*AMOTL1; LOC105369438*	11:94448263:C:T	3.0 (1.9-4.7)	4.1×10^{-6}	+++	32.4	0.23	intron variant	no significant data	5
NA	NA	11:94431032:TAAAG:T	3.0 (1.9-4.8)	4.3×10^{-6}	+++	37.3	0.20	NA	NA	NA
rs7130432	*LOC105369438*	11:94395496:A:G	0.4 (0.2-0.6)	4.3×10^{-6}	---	79.3	0.01	intron variant	significant with expression of *KDM4D* gene in vagina tissue	5

NA = Information not available.

[a] Chromosome: base pair:Allele1:Allele2

[b] Three symbols depicted the direction of association in three datasets included in the discovery cohort. The first symbol was for head and neck cancer genotyped with Illumina OncoArray (n = 254), the second symbol was for head and neck cancer genotyped with Illumina Consortium OncoArray (n = 216), and the third symbol was for esophageal cancer (n = 138). (-) protective effect; (+) risk effect; (?) results not known

Table S5. Top twenty SNPs from genome-wide meta-analysis of cisplatin-induced eGFR reduction in the discovery cohort

rsID	Genes	Chromosome:Location :Allele[a]	β	SE	P-value	Direction[b]	Heterogeneity I^2	Heterogeneity P-value	Functional Consequences	eQTL from GTEx database	RegulomeDB score
rs17161766*	*TMEM225B*	7:99177716:G:A	-28.9	5.01	7.8×10^{-9}	NA-NA	0	1	intron variant	significant with expression of *ZSCAN25, GS1-259H13.2, BUD31* in various tissues but not in kidney	4
NA*	NA	7:98951080:C:CTTAT	-27.2	4.74	9.5×10^{-9}	NA-NA	0	1	NA	NA	NA
rs199659233*	*ARPC1A*	7:98959960:T:C	28.7	5.06	1.5×10^{-8}	NA+NA	0	1	intron variant	NA	6
rs556958738*	*ARPC1A*	7:98959961:T:C	28.7	5.06	1.5×10^{-8}	NA+NA	0	1	intron variant	NA	NA
rs4388268*	*BACH2*	6:90734908:G:A	-8.4	1.52	3.8×10^{-8}	---	0	0.53	intron variant	no significant data	5
rs1826059	NA	4:64014716:A:G	7.3	1.38	1.4×10^{-7}	+++	0	0.71	NA	no significant data	No data
NA	NA	4:64016970:CTT:C	7.3	1.38	1.4×10^{-7}	+++	0	0.71	NA	NA	NA
rs62320477	NA	4:64018447:G:A	-7.3	1.39	1.5×10^{-7}	---	0	0.89	NA	no significant data	No data
NA	NA	4:63915271:A:AT	7.1	1.35	1.6×10^{-7}	+++	0	0.67	NA	NA	NA

rs6834243	NA	4:64012390:T:C	7.2	1.39	$2.3x10^{-7}$	+++	0	0.70	NA	no significant data	6
rs59242959	NA	4:64013730:G:A	-7.2	1.39	$2.3x10^{-7}$	---	0	0.70	NA	no significant data	No data
NA	NA	4:64017361:C:CTGGGTT	-7.2	1.39	$2.3x10^{-7}$	---	0	0.70	NA	NA	NA
rs62320476	NA	4:64018284:C:T	-7.2	1.39	$2.3x10^{-7}$	---	0	0.70	NA	no significant data	No data
rs62320470	NA	4:64009273:T:A	-7.2	1.39	$2.5x10^{-7}$	---	0	0.69	NA	no significant data	No data
rs138024145	NA	4:63893286:C:A	-7.0	1.35	$2.9x10^{-7}$	---	0	0.76	NA	NA	No data
rs12664728	*BACH2*	6:90737315:G:A	-10.8	2.10	$2.9x10^{-7}$	---	61.6	0.07	intron variant	no significant data	6
rs16882364	*BACH2*	6:90735491:A:G	10.7	2.09	$3.0x10^{-7}$	+++	61.8	0.07	intron variant	no significant data	2b
rs35533931	NA	4:64028203:C:T	-7.2	1.41	$3.1x10^{-7}$	---	0	0.68	NA	no significant data	No data
rs976921	NA	4:64076807:T:C	7.0	1.36	$3.2x10^{-7}$	+++	0	0.62	NA	no significant data	No data

rs2007396	NA	4:64076658:T:C	7.0	1.37	3.2×10^{-7}	+++	0	0.63	NA	no significant data	6

*reached genome-wide-significance (p-value $\leq 5 \times 10^{-8}$)

NA = Information not available.

[a] Chromosome: base pair:Allele1:Allele2

[b] Three symbols depicted the direction of association in three datasets included in the discovery cohort. The first symbol was for head and neck cancer genotyped with Illumina OncoArray (n = 254), the second symbol was for head and neck cancer genotyped with Illumina Consortium OncoArray (n = 216), and the third symbol was for esophageal cancer (n = 138). (-) reduced eGFR; (+) increased eGFR; (NA) results not known since the SNP did not surpass the post-imputation QC in that particular dataset.

Sensitivity analysis in subjects of discovery cohort with available Charlson Comorbidity Index data (*n* = 509)

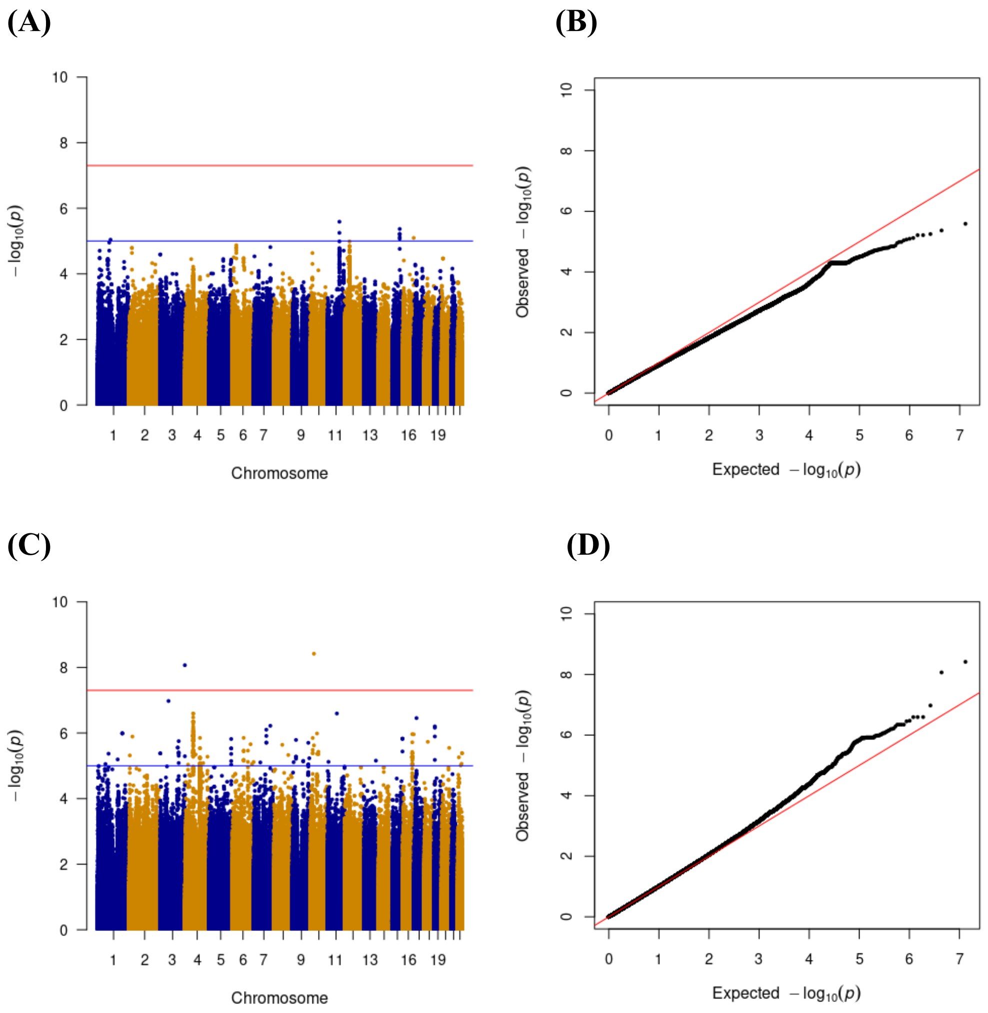

Figure S2. Genome-wide meta-analysis results of cisplatin-induced nephrotoxicity using AKI-CTCAE and eGFR phenotypes in subjects of discovery cohort with available Charlson Comorbidity Index data (n = 509). (A) Manhattan plot showing logistic regression results using the AKI-CTCAE phenotype; −log10 P-values are plotted against the respective chromosomal position of each SNP. (B) A quantile-quantile (Q-Q) plot showing the distribution of P-values in the GWAS using the AKI-CTCAE phenotype. (C) Manhattan plot showing logistic regression results using the eGFR phenotype. (D) Q-Q plot showing the distribution of P-values in the GWAS using the eGFR phenotype.

Table S6. Association between *BACH2* rs4388268 and cisplatin-induced nephrotoxicity in subjects of discovery cohort with available Charlson Comorbidity Index data (n = 509)

Chromosome: location: allele[a]	Functional consequences	Outcome	Effect size (95% CI)[b]	P-value	Direction[c]
6:90734908:G:A	Intron variant	AKI – CTCAE	3.6 (1.7 – 5.4)	$3.8x10^{-5}$	+ + +
		eGFR reduction	-8.1 (-11.4 – -4.8)	$1.4x10^{-6}$	- - -

[a] Chromosome: base pair:Allele1:Allele2

[b] OR for AKI-CTCAE phenotype and β for eGFR phenotype

[c] Three symbols depict the direction of association in the three datasets included in the discovery cohort. The first symbol is for head and neck cancer genotyped with Illumina OncoArray (n = 254), the second symbol is for head and neck cancer genotyped with Illumina Consortium OncoArray (n = 216), and the third symbol is for esophageal cancer (n = 138). For AKI–CTCAE outcome: (-) protective effect; (+) risk effect. For eGFR reduction outcome: (-) reduced eGFR; (+) increased eGFR.

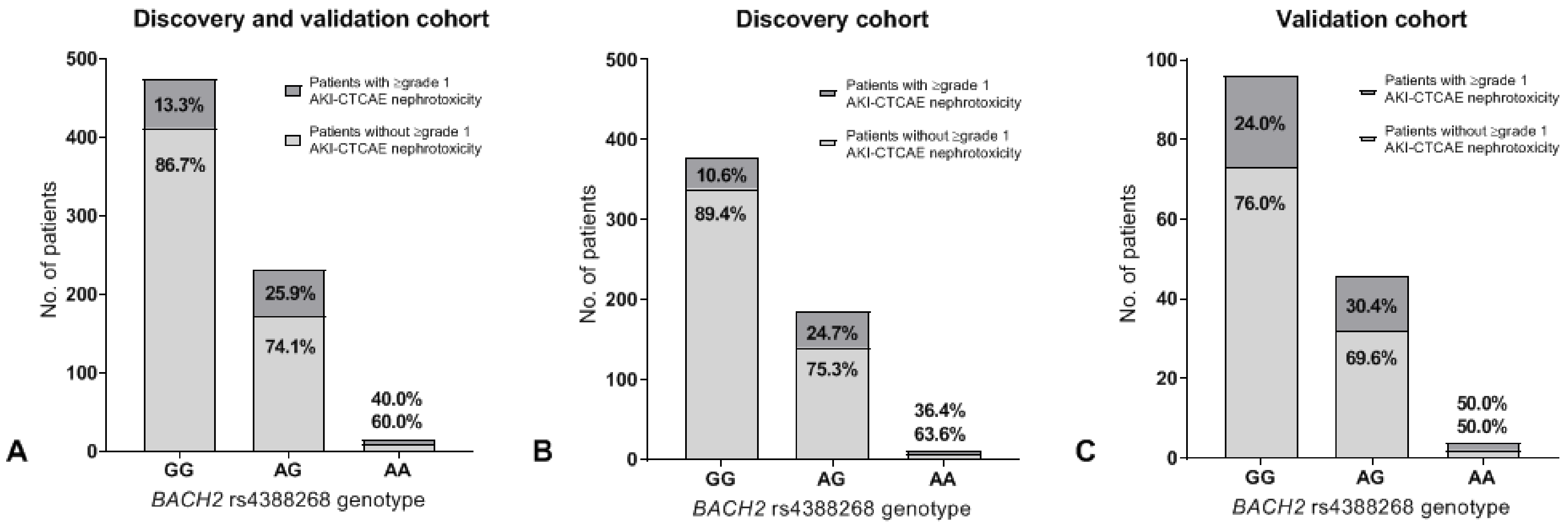

Figure S3. AKI-CTCAE status for each *BACH2* rs4388268 genotype. **A.** AKI-CTCAE status for each *BACH2* rs4388268 genotype in the overall cohort (n = 757). **B.** AKI-CTCAE status for each *BACH2* rs4388268 genotype in the discovery cohort (n = 608). **C.** AKI-CTCAE status for each *BACH2* rs4388268 genotype in the validation cohort (n = 149).

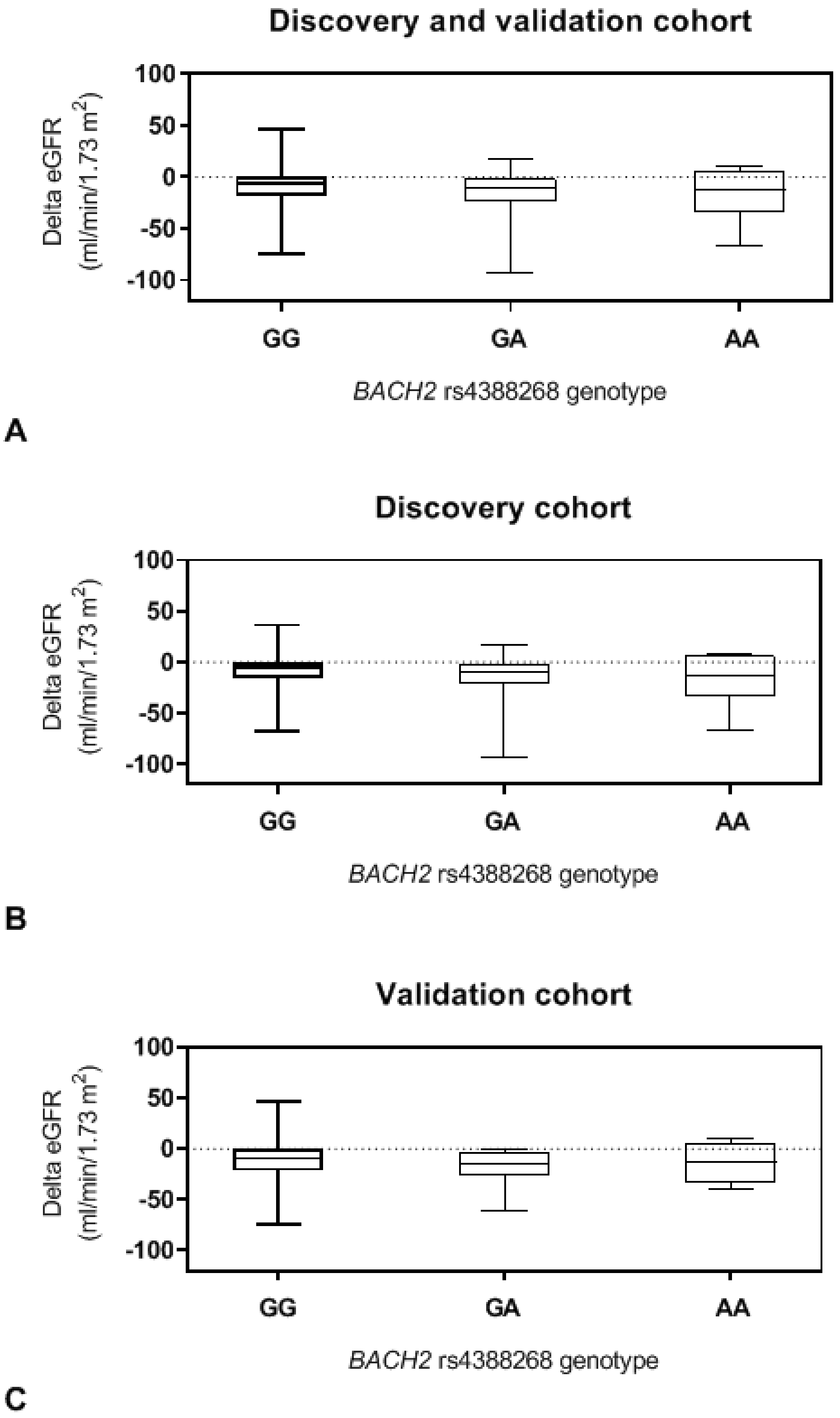

Figure S4. eGFR differences (ΔeGFR) for each *BACH2* rs4388268 genotype. **A.** eGFR differences (ΔeGFR) for each *BACH2* rs4388268 genotype in the overall cohort (n = 757). **B.** eGFR differences (ΔeGFR) for each *BACH2* rs4388268 genotype in the discovery cohort (n = 608). **C.** eGFR differences (ΔeGFR) for each *BACH2* rs4388268 genotype in the validation cohort (n = 149).

Table S7. Median of eGFR reduction for each *BACH2* rs4388268 genotype in the overall, discovery, and validation cohort

Group	GG		AG		AA	
	n (%)	Reduction of eGFR, median mL/min/1.73 m^2 (IQR)	*n* (%)	Reduction of eGFR, median mL/min/1.73 m^2 (IQR)	*n* (%)	Reduction of eGFR, median mL/min/1.73 m^2 (IQR)
Discovery and validation cohort (*n* = 757)*	475 (65.8)	6.6 (0.0 – 17.0)	232 (32.1)	9.6 (1.10 – 21.2)	15 (2.1)	13.3 (3.0 – 34.9)
Patients without nephrotoxicity (*n* = 624)	412 (66.2)	5.1 (0 – 13.7)	172 (27.7)	6.8 (0.6 – 16.7)	9 (1.4)	8.0 (7.4 – 12.9)
Patients with grade 1 or higher AKI-CTCAE (*n* = 133)	63 (47.7)	31.8 (24.2 – 44.8)	60 (45.5)	30.2 (17.2 – 38.0)	6 (4.5)	36.9 (34.5 – 44.4)
Grade 1 (*n* = 104)	49 (47.6)	29.9 (24.2 – 38.7)	46 (44.7)	27.6 (16.9 – 35.4)	5 (4.9)	34.9 (34.5 – 39)
Grade 2 (*n* = 21)	10 (47.6)	53.2 (42.8 – 56)	11 (52.4)	45.5 (15.3 – 57.6)	0 (0)	0 (0)
Grade 3 (*n* = 8)	4 (50)	63.5 (25.8 – 72.5)	3 (37.5)	66.2 (48.6 – 92.8)	1 (12.5)	66.4
Discovery cohort (*n* = 608)**	379 (65.8)	6.2 (-16.2 – 0.0)	186 (32.3)	9.6 (1.1 – 22.4)	11 (1.9)	13.3 (6.5 – 34.8)
Patients without nephrotoxicity (*n* = 515)	339 (65.8)	5.1 (1.1 – 13.7)	140 (27.2)	6.6 (0.7 – 16.7)	7 (1.4)	12.8 (7.4 – 13.3)
Patients with grade 1 or higher AKI-CTCAE (*n* = 93)	40 (43.0)	30.6 (16.4 – 42.9)	46 (49.5)	27.5 (14.0 – 42.0)	4 (4.3)	39.6 (34.7 – 55.4)
Grade 1 (*n* = 71)	33 (46.5)	30.5 (24.2 – 35.7)	32 (45.1)	26.2 (8 – 35.6)	3 (4.2)	34.9 (34.5 – 44.4)
Grade 2 (*n* = 17)	6 (35.3)	47.4 (4.9 – 54.4)	11 (64.7)	45.5 (15.3 – 57.6)	0 (0)	0 (0)
Grade 3 (*n* = 5)	1 (20)	5.5	3 (60)	66.2 (48.6 – 92.8)	1 (20)	66.4
Validation cohort (*n* = 149)***	96 (65.8)	10.0 (0.0 – 21.0)	46 (31.5)	9.0 (0.0 – 19.0)	4 (2.7)	13.5 (5.0 – 46.8)
Patients without nephrotoxicity (*n* = 109)	73 (68.2)	5.0 (0.0 – 13.0)	32 (29.9)	8.0 (0.0 – 17.5)	2 (1.9)	-1.0 (-10.0 – 8.0)
Patients with grade 1 or higher AKI-CTCAE (*n* = 40)	23 (59.0)	40.0 (26.0 – 56.0)	14 (35.9)	33.0 (26.0 – 35.0)	2 (5.1)	28.5 (18.0 – 39.0)
Grade 1 (*n* = 33)	16 (50)	28 (24.5 – 40)	14 (43.8)	33 (26 – 35)	2 (6.3)	28.5 (18 – 39)
Grade 2 (*n* = 4)	4 (100)	57 (53.5 – 60)	0 (0)	0 (0)	0 (0)	0 (0)
Grade 3 (*n* = 3)	3 (100)	70 (57 – 75)	0 (0)	0 (0)	0 (0)	0 (0)

*Missing genotype in 35 patients.

** Missing genotype in 32 patients.

*** Missing genotype in 3 patients.

Supplementary S1. Calculations number needed to genotype (NNG) and number needed to treat (NNT) on *BACH2* rs4388268 based on formula provided by Tonk, *et al*. (2017)

1. **Discovery cohort**

Genotype	With nephrotoxicity	Without nephrotoxicity
AA	4	7
AG	46	140
GG	40	339

Allele	With nephrotoxicity	Without nephrotoxicity	Total
A	54	154	208
G	126	818	944
Total	180	972	1152

Type of effect sizes	Value
RR	1.95
RD	0.13
NNT	7.93
NNG	43.91

2. **Validation cohort**

Genotype	With nephrotoxicity	Without nephrotoxicity
AA	3	1
AG	8	22
GG	14	53

Allele	With nephrotoxicity	Without nephrotoxicity	Total
A	14	24	38
G	36	128	164
Total	50	152	202

Type of effect sizes	Value
RR	1.68
RD	0.15
NNT	6.72
NNG	35.7

3. **Combined cohort**

Genotype	With nephrotoxicity	Without nephrotoxicity
AA	7	8
AG	54	162
GG	54	392

Allele	With nephrotoxicity	Without nephrotoxicity	Total
A	68	178	246
G	162	946	1108
Total	230	1124	1354

Type of effect sizes	Value
RR	1.89
RD	0.13
NNT	7.68
NNG	42.27
